# Effects of left anodal transcranial direct current stimulation on hypothalamic–pituitary–adrenal axis activity in depression: a randomized controlled pilot trial

**DOI:** 10.1038/s41598-023-32531-6

**Published:** 2023-04-06

**Authors:** Bruno Pedraz-Petrozzi, Helena Sardinha, Maria Gilles, Michael Deuschle

**Affiliations:** Department of Psychiatry and Psychotherapy, RG Stress, Central Institute of Mental Health, Medical Faculty Mannheim, University of Heidelberg, J5, 68159 Mannheim, Germany

**Keywords:** Randomized controlled trials, Stress and resilience, Predictive markers, Depression, Chronic inflammation, Hypothalamus, Pituitary gland

## Abstract

The main objective of this study was to evaluate the effect of left anodal transcranial direct current stimulation (tDCS) on hypothalamic–pituitary–adrenal axis (HPAA) activity in individuals with depression. We conducted a 3-week, randomized, triple-blind pilot trial with 47 participants (dropout rate: 14.89%) randomly assigned to either the tDCS or control group (sham stimulation). Salivary cortisol was used as an HPAA activity marker since cortisol is the effector hormone of the HPAA. The primary outcome was the effect of tDCS on the diurnal cortisol pattern (DCP and area under the curve with respect to ground -AUCg-). Secondary outcomes included tDCS effects on cortisol awakening response (CAR) and cortisol decline (CD), as well as the variation of cortisol concentrations between the initiation of tDCS and 2 weeks later. Intention-to-treat and per-protocol analyses were conducted. Our primary outcome showed an absent effect of tDCS on DCP and AUCg. Additionally, tDCS had an absent effect on CAR, CD, and cortisol concentration variation before-after stimulation. Our pilot study suggests that anodal tDCS showed an absent effect on HPAA activity in individuals with depression. More studies are needed to confirm these findings.

## Introduction

Depression is a mood disorder characterized by permanent sadness, anhedonia, loss of perspective, and suicidal behavior^1,2^. The World Health Organization (WHO) estimates that approximately 300 million people worldwide suffer from depression, resulting in a global prevalence of 4.4%, with substantial costs to regional health systems and high disability-adjusted life years^3^.

Although numerous hypotheses have been proposed regarding the causes and pathophysiology of depression, they remain uncertain. However, recent studies have revealed a correlation between depressive episodes and inflammation^4^ or high hypothalamic–pituitary–adrenal axis (HPAA) activity, which is manifested by altered diurnal cortisol patterns^5–7^, elevated morning salivary cortisol^8,9^, and increased cortisol awakening response^10^ during depression. Additionally, research has shown that pharmacological treatment leads to a reduction in HPAA activity^11^, with the modulatory effect on cortisol metabolism being a critical component for the treatment of depression.

International guidelines for treating depressive episodes typically recommend antidepressant agents combined with psychotherapeutic treatment^12,13^. However, about 15–30% of patients with depression do not respond to guideline-recommended therapies^14^. Novel augmentation treatments, such as non-invasive procedures like transcranial direct current stimulation (tDCS)^15–18^, have been developed in the last three decades to address non-response^19,20^.

The evidence linking tDCS and depression is growing, but there is a lack of research on the effect of tDCS on HPAA activity in depression, as depression itself can alter HPAA activity^21–23^. Additionally, no studies have evaluated the effect of tDCS on cortisol levels in depressed patients, despite evidence that other stimulation procedures (e.g., electroconvulsive therapy)^24,25^ and pharmacological agents (e.g., mirtazapine and tricyclic antidepressants)^11,26^ have been linked to reductions in cortisol levels and improved clinical outcomes in depressive episodes. Given the critical role of the prefrontal cortex (PFC) in regulating the HPAA stress response^27^, the hypoactivity of the PFC and the dysregulation of the HPAA observed in mood disorders, stimulation of the PFC through tDCS may increase its activity and regulate HPAA and cortisol metabolism^28^.

Therefore, the main objective of our randomized, triple-blind pilot trial was to assess the effect of tDCS on HPAA activity in depressed participants by evaluating salivary cortisol levels. We hypothesized that the tDCS would be an effective treatment for depression and reduce salivary cortisol levels in participants with depression.

## Materials and methods

### Study design

This randomized, triple-blind pilot trial was conducted at the Central Institute of Mental Health (Mannheim, Germany). The pilot trial aimed to compare the effect of tDCS on HPAA activity in patients with depressive episodes, and included an experimental group (tDCS) and a control group (sham stimulation). Diurnal cortisol pattern (DCP), area under the curve with respect to the ground (AUCg) of the diurnal cortisol pattern, cortisol awakening response (CAR), cortisol decline (CD), and variation of cortisol before and after stimulation were calculated in both groups. Pharmacological and psychotherapeutic treatments were continued throughout the trial. The trial schedule and measurements are presented in Table 1. This study is registered in the German Clinical Trials Register (DRKS; register Number ID DRKS00029994; registration date: 16/08/2022).

**Table 1:** Trial schedule and measurements (trial period = 3 weeks = 21 days).

		Stimulation trial			
Weeks (W)	Baseline	W0	W1	W2	W3
Informed consent and study information	X				
Proof of inclusion and exclusion criteria	X				
Stimulation (tDCS/sham stimulation)		X	X	X	X
*Antidepressant Treatment History Form* (ATHF)	X				
Salivary cortisol, diurnal cortisol pattern (DCP) (4 Samples: Cor I, II, III, IV)	X				X
Salivary cortisol immediately before stimulation		X		X	
Salivary cortisol immediately after stimulation		X		X	
Salivary cortisol 2 h after stimulation		X		X	
*Montgomery-Åsberg Depression Rating Scale* (MADRS)	X	X	X	X	X

tDCS, transcranial direct current stimulation; W0, first stimulation; W1, one week after first stimulation; W2, two weeks after first stimulation; W3, three weeks after first stimulation; Cor I, salivary cortisol measured between 7:30 and 8:00 AM; Cor II, salivary cortisol measured between 8:00 and 8:30 AM; Cor III, salivary cortisol measured between 3:30 and 4:00 PM; Cor IV, salivary cortisol measured between 9:30 and 10:00 PM.

### Randomization and blinding

Study participants, who were patients of an inpatient affective disorders’ unit, were randomly assigned in a 1:1 ratio to either an experimental group receiving tDCS or a control group receiving sham stimulation. To mitigate potential bias in the results, especially experimental bias, each patient was assigned to one of the four stimulation devices (A, B, C, and D) based on the order of their inclusion in the study. Two of the four devices were tDCS (A and C), and the remaining two were sham stimulation devices (B and D). To ensure compliance with the quality criterion of reliability, all stimulation treatments were carried out by the same practitioner (H.S.) using the same devices, in the same rooms, and always on weekdays (Monday–Friday) between 2:00 and 4:00 PM. The same trial physician always collected the assessment questionnaires. During the trial, neither the patients, the practitioner, nor the trial physician were informed about the devices’ mode (sham stimulation or tDCS) or whether the treatment was real or simulated. This blinding status was maintained throughout the evaluation of the raw data, and disclosure only took place at the beginning of the final data analysis to further reduce possible experimental bias.

### Participants

Initially, between August 15th, 2016 and September 20th, 2017, 47 patients (20 female and 27 male participants; mean age: 45.30 ± 14.20) with depressive disorders were recruited from the inpatient unit “affective disorders” at the Central Institute of Mental Health, Mannheim for this pilot trial. The characteristics of the participants who completed the trial are presented in Table 2. To be included in the study, participants had to meet the DSM-5 criteria for single or recurrent major depressive episode, as evaluated through SCID-I interviews, and sign the informed consent form. The interviews were conducted by clinical experts at the Central Institute of Mental Health who were not involved in the study. Patients were included if they had a Hamilton Depression Rating Scale (HDRS) score of at least 18 points with baseline stability (i.e., less than 25% improvement 1 week before the beginning of stimulation). Before starting the study, we ensured that participants had an ECG sinus rhythm. Additionally, this pilot trial did not require a therapy-naïve status, so participants could continue their prescribed treatments at the inpatient unit. We chose a three-week trial interval (21 days) to avoid the occurrence of essential changes in the pharmacological treatment. Finally, participants taking benzodiazepines as a pro re nata (PRN) treatment could participate with doses no greater than 1.5 mg/days of Lorazepam or equivalent to minimize additional pharmacological effects. Participants taking hypnotics, such as zopiclone and zolpidem, as PRN treatments were not excluded from this pilot trial.

**Table 2:** Characteristics of the participant groups.

Baseline characteristics	tDCS (n = 24)	Sham stimulation (n = 23)	p
Gender (f/m)	11/13	9/14	0.239
Age (in years)	48.29 (15.21)	42.13 (12.65)	0.181
Weight (in kg)	86.05 (17.96)	81.42 (18.07)	0.378
Edinburgh Laterality Coefficient	78.15 (41.12)	91.29 (23.98)	0.234
Number of depressive episodes (including actual episode)	3 (1.64)	3.42 (2.06)	0.478
Age at disease beginning (in years)	36.38 (13.00)	30.41 (10.61)	0.136
ATHF scores	5.89 (2.47)	5.81 (3.64)	0.931
Somatic Symptoms (y/n)*	12/9	13/6	0.462
Family history of unipolar depression (y/n)*	10/11	8/11	0.726
Main diagnosis according to DSM5 (unipolar/bipolar depression) *	19/2	16/3	0.451
First depressive episode (first/recurrent)*	4/17	3/16	0.786
Duration of current episode (months)*	4.05 (1.27)	4.05 (1.69)	0.992
Comorbid dysthymia (yes/no)*	5/16	2/17	0.412
Comorbid generalized anxiety disorder (yes/no)*	5/16	2/17	0.412
Comorbid personality disorder, other than borderline personality disorder (yes/no) *	3/18	3/16	1.000
Total MADRS Scores			
W0**	33.46 (6.32)	29.30 (6.48)	1.000
W1**	29.08 (6.04)	28.48 (4.31)	1.000
W2**	26.13 (8.49)	24.22 (7.14)	1.000
W3**	21.79 (7.39)	19.00 (7.81)	1.000

Values are expressed as mean (standard deviation).

W0, first stimulation; W1, one week after first stimulation; W2, two weeks after first stimulation; W3, three weeks after first stimulation; ATHF, Antidepressant treatment history form; DSM-5, Diagnostic and Statistical Manual of Mental Disorders, fifth version; MADRS, Montgomery–Åsberg depression rating scale; tDCS, Transcranial direct current stimulation.

*Of the 47 patients included in the study, 40 completed the three-week treatment, and seven dropped out of the study before finishing the trial. Hence, data of 4 participants of the sham stimulation group and 3 of the tDCS are missing.

**p-values are Bonferroni-corrected.

Patients with psychotic disorders, post-traumatic stress disorders, panic disorders, and borderline personality disorders were excluded from the study. We also excluded pregnant female participants, participants with a conservatorship or legal guardianship, and patients who were unable to provide consent due to severe mental illness. Furthermore, patients who met the following criteria were excluded: those with metal implants or medical devices (e.g., cardiac pacemakers), those with illegal drug and alcohol dependency at the time of the study, those experiencing acute suicidal crisis, those with medical conditions that alter adrenal functions, those taking glucocorticoids or medications that alter heart rate and variability (such as beta-blockers), those with any type of arrhythmia, those meeting DSM-5 criteria for dementia syndrome, those with a past medical history of severe cranioencephalic trauma, those with any neurological disease, those with severe decompensated medical conditions (such as therapy-resistant arterial hypertension, heart insufficiency, or respiratory insufficiency), and those with neoplastic or infectious diseases of any kind.

Of the 47 patients initially included in the study, 40 completed the three-week treatment, resulting in a dropout rate of 14.89%. One 20-year-old participant was interrupted on the third day of stimulation due to a newly identified skin rash on the scalp, and a 78-year-old participant was discharged from the inpatient ward, interrupting the trial on the seventh day. Three participants (ages 34, 48, and 53) were terminated from the trial before the first stimulation appointment due to new diagnostic assessments, resulting finally in exclusion. Additionally, one participant (age 38) left the study before the first stimulation appointment due to the onset of a manic phase. Lastly, one participant (age 27) withdrew from the pilot study due to post-stimulation headaches.

Regarding medication, all 40 participants (35 participants with unipolar depression and 5 participants with bipolar depression) who completed the stimulation trial received direct current stimulation as an add-on therapy to guideline-compliant pharmacotherapy for depression. Among them, seven received monotherapy with an antidepressant agent (SSRI, SNRI, bupropion, and trazodone), 14 were treated with two antidepressants (SSRI + mirtazapine, SSRI + trazodone, bupropion + trazodone), and one with a combination of three antidepressants (bupropion + mirtazapine + trazodone). Six patients were prescribed an antidepressant plus augmentation therapy (such as aripiprazole, quetiapine, or lithium), and five received a combination of two antidepressants and augmentation therapy. One patient received a quadruple combination of three antidepressants and quetiapine, and two received a triple combination of lithium, quetiapine, and an antidepressant. Two patients received quetiapine monotherapy, and two received dual therapy with lithium and quetiapine. There were no significant differences between the two groups concerning pharmacotherapy (Cramer’s V = 0.70, p = 0.42).

### Transcranial direct current stimulation (tDCS)

Stimulation was administered using a CE-certified microprocessor-controlled device (Sooma tDCS, Sooma Medical, Helsinki, Finland) that emitted a direct current (DC) with a maximum output of 2 mA. A pair of conductive rubber square electrodes (anode and cathode) with dimensions of 5 × 7 cm (35 cm^2^; ELM2 Electrodes, Sooma Medical, Sooma Oy, Helsinki, Finland) were used to apply DC. Prior to electrode placement, tDCS electrode sponges (50 × 70 mm; SPM200, Sooma Medical, Sooma Oy, Helsinki, Finland) were soaked in a 144 mol/L NaCl solution to lower the physiological skin resistance. The electrodes were placed based on the 10–20 international system, with the anode positioned at EEG point F3 (left dorsolateral PFC) and the cathode at EEG point Fp2 (right dorsolateral PFC). Correct placement was facilitated by a hood (Large/Medium/Small Flex cap, Sooma Medical, Sooma Oy, Helsinki, Finland) and chin strap (HCHI, Sooma Medical, Sooma Oy, Helsinki, Finland), adapted to the head of the participant. The cap was pulled taut with the edge ending approximately 1.5–2 cm above the eyebrows and positioned correctly. The chin strap was then secured in place.

The four stimulation devices (A, B, C, and D) were preprogrammed to provide either real stimulation (devices A and C, with a DC of 2.00 mA) or sham stimulation (devices B and D, with a DC of 0.30 mA). In the sham stimulation mode, the devices initially provided DC of 2.00 mA, immediately ramping down to a DC of 0.30 mA and maintaining this current for 30 min (treatment duration), thereby producing sensations similar to real stimulation.

Stimulation was carried out according to the manufacturer’s protocol, which entailed three weeks of treatment with daily 30-min stimulation sessions for 15 sessions. This protocol was used irrespective of the device mode (real or sham stimulation). In our trial, participants received tDCS or sham stimulation until the end of week 3, from Monday to Friday, between 2:00 and 4:00 PM, with stimulation pauses at weekends.

### Cortisol assessment

#### General procedure

The activity of the HPAA was assessed by measuring cortisol in saliva samples. Salivary cortisol was preferred due to its practicability in sample collection, which is less invasive and requires less effort from participant compared to other methods^9,29^. Studies have demonstrated a good correlation between salivary and plasma cortisol^30,31^, verifying its effectiveness for assessing plasma cortisol concentration. Salivary cortisol measurements were acquired as reliable indicators of total free plasma cortisol, albeit with marginally lower concentrations due to the presence of 11β-HSD2 in saliva^32^.

Diurnal cortisol data were obtained using Salivette® tubes (Sarstedt™, Leicester) containing an untreated cotton swab. Four saliva samples were collected from participants on a typical ward routine day. The participants were all inpatients on the same ward with identical routines. Participants were instructed to chew on the saliva collectors after awakening (Cor I), 30 min after getting up (Cor II), 8 h after awakening (Cor III), and 14 h after awakening (Cor IV), while still in bed. Additional instructions were provided by ward health workers (i.e., nurses, postgraduate trainees or interns) regarding precautions related to meals, drinks, teeth brushing, and smoking. The ward health workers also recorded the date and time of sampling and placed the tubes with saliva samples in the refrigerator. All samples were stored at – 25 °C, and after thawing, they were centrifuged for 5 min at 3000 rpm, resulting in a clear supernatant of low viscosity. Salivary cortisol levels were measured using a time-resolved immunoassay with fluorescence detection^33,34^. The inter- and intra-assay coefficients of variation were less than 10% across the expected range of cortisol levels, and the lower limit of detection was 0.43 nmol/L. The concentrations were calculated in nmol/L^33,34^.

#### Saliva samples for DCP, AUCg, cortisol awakening response, cortisol decline, and stimulation-related cortisol levels

Two sets of DCP samples were collected during the study. Participants were instructed to collect saliva four times during the day before the first stimulation (baseline) and at the end of the third week (W3)^35^. Additionally, the area under the curve with respect to the ground (AUCg) was calculated based on the DCP data using the formulae of Pruessner et al.^36^. AUCg reflects the total cortisol concentration over the course of a day, as measured by the area under the diurnal cortisol curve using a trapezoidal formula.

Based on samples Cor I and Cor II, the cortisol awakening response (CAR) was calculated from the difference between the two samples (i.e., CAR = Cor II−Cor I). Additionally, the cortisol decline (CD) was calculated from the difference between the first morning cortisol value (i.e., sample Cor I) and the last value (i.e., sample Cor IV). Finally, salivary cortisol was collected "immediately before stimulation" (pre), "immediately after stimulation" (post), and "2 h after stimulation" (2 h-post) in W0 and W2 (Table 1) to record the short-term effects of tDCS on HPAA activity.

It is important to note that the examination of diurnal cortisol parameters (i.e., CAR, CD and DCP) has been established as an important aspect in both clinical and epidemiological research^37^. In addition, recent studies highlight the significance of diurnal salivary cortisol profiles in understanding adrenocortical functioning and regulation of the HPAA. The relevance of diurnal cortisol parameters, including CAR, CD, and DCP, is also due to the fact that cortisol is a hormone that displays a circadian rhythm and varies throughout the day, and the level of salivary cortisol changes depending on the time of day of the analysis^37^.

Implausible morning cortisol values (i.e., values of samples Cor I or Cor II ≤ 3 nmol/L) were replaced with half of the minimum morning cortisol value (in this case, 1.50 nmol/L), as recommended elsewhere^38^. In our case, five replacement procedures for four participants were required, with four participants having a Cor I value less than or equal to 3 nmol/L and being replaced with a value of 1.50 nmol/L. One participant had both Cor I and Cor II values below 3 nmol/L, and their data were also replaced with a value of 1.50 nmol/L.

### Outcomes

As the main objective of this pilot trial was to assess the effect of tDCS on HPAA activity in depressed participants by evaluating salivary cortisol levels, we defined our primary outcome as the detection of any changes in the DCP from the baseline to W3 in the tDCS group compared to the control group, including also changes in AUCg for salivary cortisol. Moreover, our secondary outcomes comprised changes in CAR and CD from baseline to W3, as well as changes in cortisol levels associated to stimulation between W0 and W2.

We also examined any variations in the Montgomery-Asberg Depression Rating Scale (MADRS) scores throughout the trial period for both groups. Following the recommendation of previous studies^39^, we defined clinical response as a reduction of ≥ 50% in the MADRS total values compared to the initial value, and remission as a final score of ≤ 9 points. Finally, also as described elsewhere^40^, we defined partial response if the MADRS total values showed a reduction of ≥ 25% of the initial value.

### Statistical analysis

Numeric variables that followed a gaussian distribution are presented as mean (standard deviation), while those with a non-gaussian distribution (i.e., median ≠ mean) are shown as median (interquartile range, 3rd quantile–1st quantile). Category variables and count data are specified as numbers or fractions, with values rounded to two decimals. Values smaller than 0.005 are presented as < 0.005, and values greater than 1 million are expressed in scientific notation^4^. Descriptive data are presented in tables. For significance testing, t-tests were used for continuous gaussian distributed data, while the U-test for continuous non-gaussian distributed data. The χ^2^ or the Cramer’s V for the category and/or count data. Statistical significance was defined as a two-tail-p-value of less than or equal to 0.05.

Primary and secondary outcomes, as well as MADRS scores, were analyzed blindly by one of the authors (B.P.P) who did not know the group assignments. Both intention-to-treat (ITT) and per-protocol analyses (PP) were performed using linear mixed models (LMM) for the primary and secondary outcomes. We calculated the interaction *time* * *daytime* * *group* (DCP) and *time* * *group* (AUCg) for the primary outcome, and *time* * *group* (CAR and CD) and *time* * *daytime* * *group* (stimulation-related cortisol levels) for the secondary outcome.

Interactions were corrected for *gender*, *age,* and *weight* as confounding factors, with multiple imputations with linear interactions and a maximum of 1000 iterations in case of missing values. For both trial groups, CAR, DCP, AUCg, CD, and stimulation-related cortisol levels were estimated in the multiple imputations using the variables gender and age of the participants. In the LMM, fixed effect omnibus tests were carried out to define the main effects of the variables in the model and to evaluate the variable in the model against the null model. The ITT results were reported graphically, and both ITT and PP analyses’ results were described in the text using 95% confidence intervals (95CI). Cohen’s d was calculated for ITT and PP analyses to estimate the sample size effect. Post-hoc tests were performed for the LMM when differences between the *time* * *group* (CD, CAR, AUCg) and the *time* * *daytime* * *group* (DCP and stimulation-related cortisol levels) were significant.

Statistical analyses and descriptive data were conducted using IBM SPSS Statistics software, version 26.0 (International Business Machines Corporation, New York, United States of America). LMM analyses were performed using the R-based software jamovi 2.0.0^41^ and the GAMLj toolbox^42^. Graphs were generated using Prism 8 GraphPad software (GraphPad Software Inc., California, United States of America).

### Ethics approval and consent to participate

Each participant was fully informed about the objectives and procedures of the study, as well as the potential adverse effects, and gave their written consent to participate. The study protocol and all study procedures were reviewed and approved by the ethics committee of the Medical Faculty Mannheim of the University of Heidelberg. Additionally, this pilot trial was carried out according to the Helsinki Declaration.

## Results

### MADRS scores during the trial

**Figure 1:**
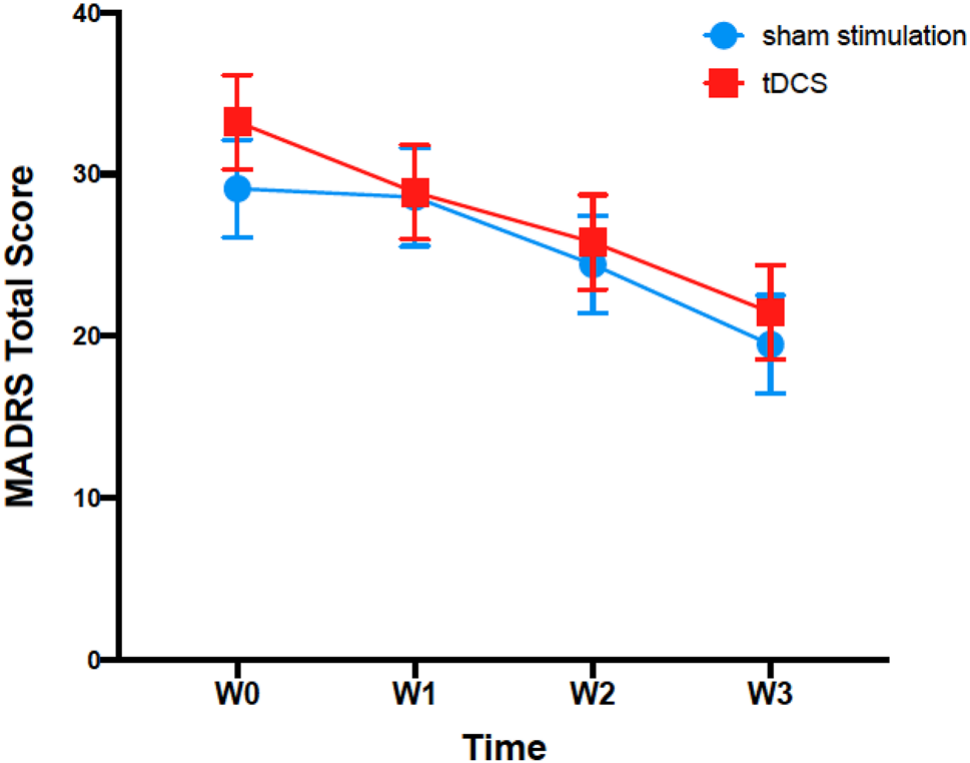
MADRS total score as response variable; estimated marginal means for MADRS scores for tDCS and sham stimulation groups.

The ITT analysis for MADRS scores revealed a significant decrease in the total MADRS score of 10.68 points (34.26% reduction from baseline scores) after 3 weeks, regardless of the trial group (95CI [− 12.92; − 8.44]; *Cohen’s d* = − 1.65). Although reductions in MADRS scores occurred in both groups, the ITT analysis showed absent effects for the interaction *time* * *group* (percentage of responders in sham stimulation group: 34.78%, percentage of responders in the tDCS group: 29.17%; Fig. 1). Interestingly, all four remitters in the study belonged to the placebo group. Finally, the PP analysis also showed absent effects for the interaction *time* * *group* but significant changes in the MADRS values during the trial time.

### Adverse effects

Adverse effects resulting from tDCS or sham stimulation were recorded using a self-assessment questionnaire at W0. These adverse effects included itching, tingling, burning, cutaneous rash, pain (χ^2^_1,40_ = 0.83, p = 0.362), concentration deficit (absent in all participants), acute mood changes (χ^2^_1,40_ = 0.17, p = 0.679), and visual perception disorders (χ^2^_1,40_ = 0.17, p = 0.679). A statistically significant difference between the two stimulation groups was only observed for the side effect of tingling (χ^2^_1,40_ = 4.96, p = 0.026). While only 6 of 13 participants in the sham group reported tingling as a side effect, this side effect occurred in 15 of the 21 participants in the tDCS group. The side effects of itching and burning were also reported more frequently by the tDCS group; however, the differences between the two groups concerning itching (χ^2^_1,40_ = 0.47, p = 0.492) and burning (χ^2^_1,40_ = 0.59, p = 0.444) were not significant. Four patients treated with tDCS and one with sham stimulation reported a cutaneous rash after stimulation, but differences between the groups were not significant (χ^2^_1,40_ = 1.43, p = 0.233). Finally, no occurrence of a manic clinical condition was observed in any test person, either under sham stimulation or under tDCS.

### Primary outcome

**Figure 2:**
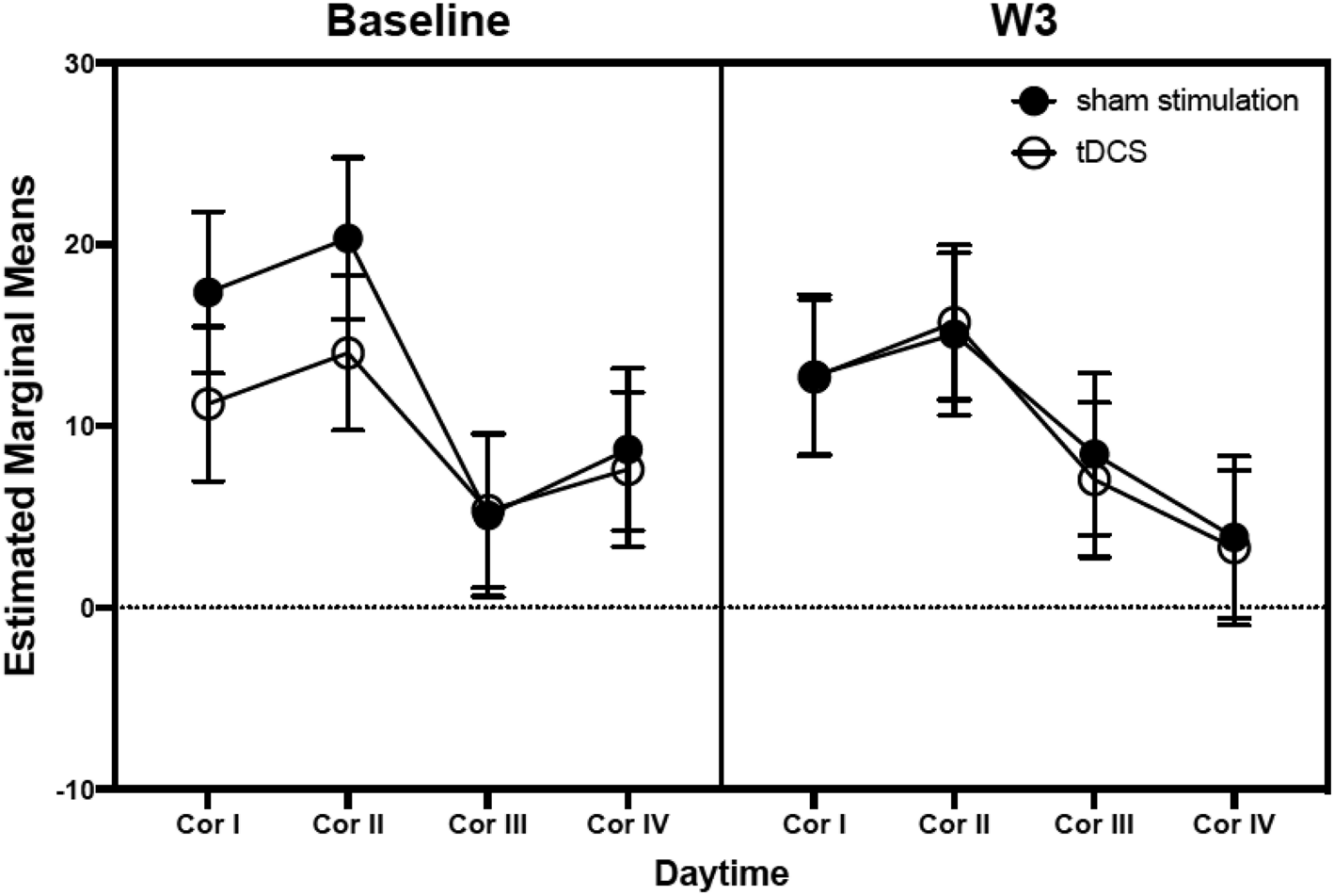
Estimated marginal means of diurnal cortisol profile (nmol/L) for tDCS and sham stimulation groups. Cor I, between 7:30 and 8:00 AM; Cor II, between 8:00 and 8:30 AM; Cor III, between 3:30 and 4:00 PM; Cor IV, between 9:30 and 10:00 PM.

The ITT analysis showed a decrease in salivary cortisol concentration during the day, regardless of the trial group (tDCS and sham stimulation) or stimulation protocol time (baseline and W3), with a decrease of 7.92 nmol/L cortisol during the course of the day (95CI [− 10.73; − 5.12]; *Cohen’s d* = − 0.64). Cortisol concentrations regarding diurnal cortisol patterns are represented in Table 3. However, we found an absence of effects for group (tDCS and sham stimulation), the interaction *daytime* * *time* (baseline and W3), or the interaction *group* * *daytime* * *time* (estimate = − 5.57, 95CI [− 16.50; 5.67]; *Cohen’s d* = − 0.11; Fig. 2). Additionally, for AUCg, the ITT analysis showed an absence of effect for *group* * *time* (estimate = 24.06, 95CI [− 34.23; 82.65]; *Cohen’s d* = 0.24).

**Table 3:** Characteristics of the participants’ experimental concentrations of cortisol.

	tDCS (n = 24)	Sham stimulation (n = 23)	p
DCP (nmol/L)			
Baseline			
Cor I**	11.19 (8.59)	16.42 (15.20)	1.000
Cor II**	13.94 (7.54)	20.36 (10.19)	1.000
Cor III**	5.32 (4.84)	5.14 (1.97)	1.000
Cor IV**	7.64 (19.46)	7.46 (19.94)	1.000
W3			
Cor I**	12.63 (5.41)	13.15 (8.35)	1.000
Cor II**	15.68 (8.34)	15.60 (9.81)	1.000
Cor III**	7.10 (5.51)	8.51 (4.58)	1.000
Cor IV**	3.24 (3.73)	3.66 (3.44)	1.000
Stimulation-related cortisol concentrations (nmol/L)			
W0			
Pre**	5.42 (3.11)	5.97 (3.66)	1.000
Post**	3.87 (3.19)	4.93 (3.11)	1.000
2 h-post**	3.66 (2.42)	3.27 (2.33)	1.000
W2			
Pre**	6.20 (3.21)	3.33 (3.53)	0.307
Post**	5.37 (2.65)	2.77 (3.19)	0.822
2 h-post**	6.33 (5.19)	4.36 (2.83)	1.000
Cortisol decline (nmol/L)			
Baseline	7.22 (12.49)	9.88 (20.22)	1.000
W3	9.53 (7.01)	9.82 (8.91)	1.000
Cortisol awakening response (nmol/L)			
Baseline	3.01 (6.23)	3.26 (15.03)	1.000
W3	3.05 (10.74)	2.45 (6.10)	1.000
Area under the curve with respect to the ground (AUCg)			
Baseline	122.21 (73.03)	148.97 (99.62)	0.975
W3	129.18 (64.79)	137.52 (47.57)	1.000

Values are expressed as mean (standard deviation).

Pre, immediately before stimulation; post, immediately after stimulation; 2 h-post, 2 h after stimulation; Cor I, between 7:30 and 8:00 AM; Cor II, between 8:00 and 8:30 AM; Cor III, between 3:30 and 4:00 PM; Cor IV, between 9:30 and 10:00 PM.

*Of the 47 patients included in the study, 40 completed the three-week treatment, and seven dropped out of the study before finishing the trial. Hence, data of 4 participants of the sham stimulation group and 3 of the tDCS are missing.

**p-values are Bonferroni-corrected.

In the PP analysis, we observed a decrease in salivary cortisol concentration during the day, with a decrease of 8.85 nmol/L cortisol during the course of the day (95CI [− 11.72; − 5.98]; *Cohen’s d* = − 0.72). Similar to the ITT analysis, we found an absence of effects for the interactions *group* * *daytime* * *time* and *daytime* * *time*. Furthermore, for AUCg, the PP analysis showed an absence of effect for *group* * *time.*

### Secondary outcomes

**Figure 3:**
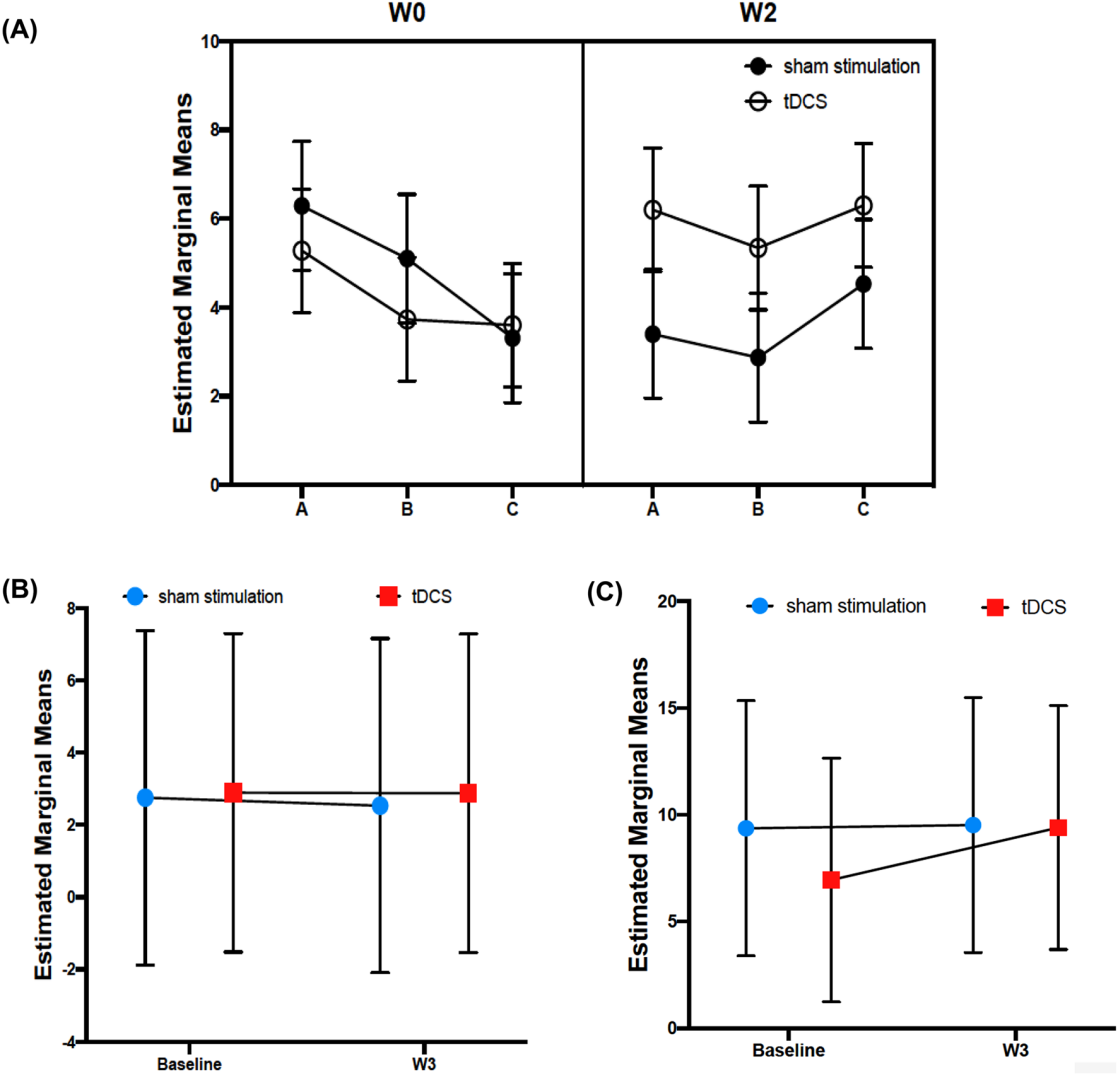
Secondary outcomes; estimated marginal means (cortisol concentration in nmol/L) were calculated in each model. (**A**) Presents the stimulation-related cortisol concentrations for tDCS and sham stimulation groups. Here, we present cortisol concentrations immediately before stimulation (**A**), immediately after stimulation (**B**), and two hours after stimulation (**C**) for W0 and W2 (Table 3). (**B**) Presents the cortisol awakening response (CAR) of both tDCS and sham stimulation groups for baseline and W3. Finally, (**C**) presents cortisol decline for both tDCS and sham stimulation groups for baseline and W3.

In the ITT analysis of secondary outcomes, there were absent effects for the interaction *group* * *time* and CAR (estimate = 0.14, 95CI [− 5.16; 5.44]; *Cohen’s d* = 0.01; Fig. 3B). Similarly, CD values also showed absent effects in the ITT analysis for the interaction *group* * *time* (estimate = 1.63, 95CI [− 4.86; 8.11]; *Cohen’s d* = 0.15; Fig. 3C). Finally, we performed an ITT analysis for the stimulation-related cortisol levels, taking into account the *trial time* (i.e., for W0 and W2) and the *stimulation time* (pre, post, and 2 h-post). However, the ITT analysis showed absent effects for the interaction *group* * *stimulation time* * *trial time* (estimate = − 2.33, 95CI [− 5.83; 1.18]; *Cohen’s d* = − 0.17 Fig. 3A). Table 3 represents cortisol concentrations related to CAR, CD, and stimulation.

In the PP analysis for CAR and CD, there were also absent effects for the interaction *group* * *time*. Additionally, the PP analysis of stimulation-related cortisol concentrations showed absent effects for the interaction *group* * *stimulation time* * *trial time*.

## Discussion

To our knowledge, this pilot study is the first to report results regarding the effects of tDCS on HPAA activity in patients with depression^43^. Regarding our primary outcome, our main findings indicate that tDCS has an absent effect on DCP in depression and absent effects in diurnal cortisol patterns (AUCg). In particular, the interaction *group* * *daytime* * *trial time* showed absent effects for the tDCS group.

Currently, there are no clinical studies evaluating the effects of tDCS on HPAA activity in participants with depression, and the current evidence regarding tDCS and HPAA activity is inconclusive. Previous studies with healthy humans have reported negative results regarding tDCS and HPAA activity^44,45^. In addition, two studies with participants with Sjogren’s disease^46^ and with osteoarthritis of the knee^47^ found no significant effects of tDCS on HPAA activity. However, previous literature, such as the review by Castelo-Branco and Fregni, based on two studies with left anodal neurostimulation in healthy subjects^28,48^, suggested that the neurostimulation of the left dorsolateral PFC (DLPFC) can regulate cortisol concentrations and cause changes in HPAA activity^49^. Finally, a systematic review by Vignaud and colleagues reported that a single stimulation of the DLPFC, independent of the stimulation procedure (i.e., repetitive transcranial magnetic stimulation -rTMS- or tDCS), is the most appropriate method to reduce cortisol concentrations in acute stress situations in healthy subjects^50^.

Despite anodal stimulation of the left DLPFC being part of our study protocol, we observed absent effects of tDCS on HPAA activity in participants with depression, in contrast to other stimulation procedures like repetitive transcranial magnetic stimulation (rTMS), which showed effects in cortisol concentration^28,51^. This suggests that tDCS may lack the necessary effects to modify HPAA activity in depression, unlike rTMS. However, given the small sample size and the pilot nature of this study, further research is needed to confirm these findings.

Regarding the secondary outcomes of CAR and CD, we found absent effects of tDCS on awakening response and decline in participants with depression. To the best our knowledge, this pilot study is the first to report on the effects of tDCS on CAR and CD in depression^43,52^. Our results suggest that tDCS, similar to the DCP, has an absent effect for inducing changes in these variables. Other evidence has also shown negative results for the effect of tDCS on CAR in healthy subjects^27^. In contrast, other stimulation procedures, such as rTMS^53^, have demonstrated significant changes in the CAR of depressive patients, which suggests that, like DCP, tDCS lacks effects to change the awaking response in people with depression. Nevertheless, larger studies are needed to confirm this finding. Although CAR is dependent on other variables, such as *age*, *gender*, and *BMI*^27,54,55^, our results were adjusted for these potential confounding factors in both analyses, reducing the likelihood of their interaction in the analysis. Lastly, as tDCS showed absent effects in the DCP, it is expected that the tDCS did not modify the CD, which is obtained by subtracting the last value from the second value of the diurnal cortisol pattern.

In terms of the stimulation-related cortisol levels, tDCS showed absent effects for the interaction *group* * *trial time* * *stimulation time* in patients with depression. Comparable negative results were also reported in a study involving healthy subjects, which revealed that tDCS did not change the cortisol concentrations associated with stimulation^27^. However, the tDCS group in our pilot trial exhibited increased cortisol concentrations (Fig. 3A). One possible explanation for the increase in cortisol levels could be the differences in adverse effects (i.e., tingling) between tDCS and sham stimulation, as well as participants’ perceived excitement levels before and during the stimulation.

As for therapy response (MADRS total scores), we noted a reduction in the MADRS score during the trial, regardless of the group assignment. This implies the effectiveness of the pharmacological treatment, while the add-on stimulation treatment showed absent effects on the MADRS scores. One possible interpretation for the lack of differences between the two stimulation procedures is that the sham stimulation could be viewed as an “active placebo”. Some studies have suggested that even lower doses may have therapeutic efficacy, as seen in the case of 0.3 mA dose applied in the sham tDCS^56^. Therefore, it cannot be entirely ruled out that the sham tDCS had therapeutic effects on patients with depressive episodes. Similar findings are reported elsewhere in the literature^57^. Finally, compared to other neurostimulation methods like rTMS, tDCS is considered a weak stimulation procedure at 1–2 mA. This may explain the lack of effects found between the study groups in this pilot trial.

This pilot trial is the first to report data on the effects of tDCS and HPAA in participants with depression. While the obtained data are negative results^58^, it is important to consider the limitations of this pilot trial. First, the initial sample size was small, with only 47 participants, and only 40 completed the trial. Larger studies would be necessary to make reliable conclusions concerning the effects of tDCS on HPAA activity in depression. Nonetheless, the scope of our study was consistent with that of other tDCS studies. Secondly, there was heterogeneity in the participants included in this pilot study. However, the two groups did not differ in demographic and clinical characteristics. Third, our study included patients with bipolar depression, as in similar studies where patients with unipolar and bipolar depression as the main diagnosis were included^59,60^. In our case, bipolar patients (n = 5) included in the study did not differ from the unipolar patients in terms of demographic or clinical characteristics. Fourth, we included participants receiving other therapy in parallel during the pilot trial. Concomitant antidepressant medication has been shown to potentially influence tDCS^61,62^ and HPAA activity^11,63–65^. However, in our study, the inclusion of the “psychopharmacological treatment” variable had an absent effect on the ITT and PP analysis results. Fifth, the timing of cortisol sampling is crucial to achieving a reliable reproduction of diurnal cortisol^66^. Another concern is the computation of cortisol indices (decline and AUCg), which were estimated using rough mathematical approximations due to the low number of daily measurements. Finally, the tDCS stimulation positioning was restricted in our study to the left anodal stimulation of the DLPFC, a common standardized protocol, but showed absent effects on the HPAA activity in depression.

In conclusion, this pilot trial showed an absent effect of a 3-week tDCS regime on the HPAA activity in depression. Future studies should consider a larger sample of possibly therapy-naïve participants and compare with other neurostimulation methods, like rTMS^68^. Moreover, exploring stimulation of other brain areas, such as the ventromedial PFC, which are linked to stress, may provide insights into the effect on HPAA activity in depression, as suggested by Carnevalli et al.^27^. Additionally, to address intraindividual variation in rhythmicity, future studies may consider collecting multiple daily measurements at various time points for more accurate reproduction of the diurnal cortisol profile and computed indices^67^. Finally, including a follow-up period in future studies could provide insight into any potential post-treatment effects (including adverse effects from repeated tDCS sessions) of tDCS on HPAA activity in depression, especially in larger studies.

## Data Availability

The data sets generated and analyzed during the study are not publicly accessible due to the applicable data protection law of the State of Baden-Württemberg but they are available from the corresponding author on justified request.
